# The effect of camel milk curd masses on rats blood serum biochemical parameters: Preliminary study

**DOI:** 10.1371/journal.pone.0256661

**Published:** 2021-09-29

**Authors:** Fatima Dikhanbayeva, Elmira Zhaxybayeva, Zhuldyz Smailova, Arman Issimov, Zhechko Dimitrov, Unzira Kapysheva, Nidhi Bansal

**Affiliations:** 1 Faculty of Food Production, Almaty Technological University, Almaty, the Republic of Kazakhstan; 2 Research and Development Centre LB-Bulgaricum PLC, Sofia, the Republic of Bulgaria; 3 School of Agriculture and Food Sciences, the University of Queensland, Brisbane, Queensland, Australia; 4 Institute of Engineering and Technology, Kyzylorda State University named after Korkyt ata, Kyzylorda, the Republic of Kazakhstan; 5 Sydney School of Veterinary Science, Faculty of Science, the University of Sydney, Sydney, New South Wales, Australia; 6 Laboratory of Ecological Physiology, Institute of humans and Animal Physiology, Almaty, the Republic of Kazakhstan; University of Agriculture in Krakow, POLAND

## Abstract

This study aimed to assess potential feeding effect of camel milk curd mass and its mixes to experimental rat’s blood serum biochemical parameters, enzymatic activity and the peptide toxicity. Fifty healthy male Sprague-Dawley rats were divided into five groups (n = 10 each). Each group was fed with camel milk pure curd mass and its mixes for 16 days. At the end of the experiment, rats were sacrificed to collect the samples from the blood serum. Blood serum biochemical parameters total protein, cholesterol, glucose, albumin, triglycerides; the enzymatic activities of alanine aminotransferase, aspartate aminotransferase, alkaline phosphatase were determined on the A25 automatic analyser, and peptide toxicity analysed by the reference method. The statistical data have shown no significant differences in body weight gain in all groups. Total protein decreased in group II, IV, and V; however, it increased in group III compared to the control group. Cholesterol grew up in group II and it slightly increased in group V, dropped in groups III and IV compared to group I result. Glucose increased in groups II, III, IV compared to group I; still, group V results show a slight decrease. Albumin decreased in group IV, yet in group V it increased than the group I result. Simultaneously, groups II and III results were changed with less percentage. Triglyceride grew up in groups II, V, and it dropped significantly in groups III, IV compared to the control group. De Ritis ratio of enzymes in groups II, III, and IV fluctuated between 1.31 and 0.98 IU/L; however, group V demonstrated significant data versus group I. Diets peptide toxicity in all groups was lower than control group data. The experimental results indicated that curd mass from camel milk could be used as a pure or with additives and it did not discover the observed side effects.

## 1. Introduction

The human body naturally tends to degrade with time, and ageing leads to adverse effects on the organism: lowered muscular body composition and growing fat mass [1], slowing the brain activity that cause Alzheimer’s [2]. The immune system is progressively weakening and endangered to cardiovascular system diseases [3] and cancer cell growth [4]. Moreover, the metabolism in the human body is dropping [5], and in some specific circumstances, long wrong nutritional habits cause dyslipidemia [6]. Those vulnerable adults who have osteoporosis and arthritis while injured are experience more sustained recovery of fractured and debilitated bones and movable joints [7]. More significant evidence and observation of elderly adults have influenced sensory perceptions of food intake, and excessive salivation was deteriorate oral health and downgraded absorption of nutrients. Merely plenty of specific cases when the elderly experience lactose intolerance, GIT dysfunctions [8], and abnormal stomach fluid increase [9]. Thus, the above listed ageing human body degradations distinctly affected by dietary habits throughout our lifetime. If adequately addressed, balanced nutrition leads to better outcomes and quality of life for mature people [10].

Over the past two centuries, a comprehensive range of scientific and research work on the study of eating behavior and the correct construction of a diet for the elderly has been conduct [11]. The successful outcomes of these academic studies’ classification of food products into the specific categories of “Geroprotectors” exhibit particular characteristics that consider specific pros and cons. These products are also obtained from dairy products, especially from fermented milk products, and they are recommended for their easy digestibility and much nutritional value. The most recommended types of sour milk products for older adults in common are yogurt, curd mass, bio-drinks, cheese (Australian Dietary Guidelines, 2020). Various nutritional components present in camel milk objectively compare to cow’s milk [12]. There are ample studies have been done on the nutritional and medicinal properties of camel milk. Many reports have shown that consumption of camel milk by diabetes patients on a daily basis reduces blood sugar and glycosylated hemoglobin (HbA_1C_) levels and also reduces insulin requirements. Despite that these findings provide scientific evidences of anti-diabetic activities of camel milk, research is yet to be initiated with assurance for patients of diabetes and other metabolic disorders. To clarify this issue, recently, there was published another study: a review, which summarizes the medicinal values of bioactive constituents of camel milk and reviews camel milk findings from the most significant preclinical studies in diabetes [13]. In addition, there is presented another study regarding diabetes and camel milk on animal model, which aimed to evaluate the antidiabetic and hepatoprotective effects, as well as lipid profile restoration of camel milk in the diabetic mouse model with duration of 7 week. As a result, scientists suggested that the camel milk could be used as a proper alternative treatment regimen for diabetes therapy [14]. Different work was investigated as a review about camel milks nutritional, antimicrobial and medicinal properties. This study authors included more significant materials regarding camel milk [15]. The one study conducted with autistics rats investigated the effects of camel milk on their antioxidant activity and enzymes. According to their received results, camel milk could recover the valporic acid induced impairment of social interaction and repetitive behaviors in the autistic rats and improve the defects in their antioxidant defense system [16]. Other research work identified that [17] the sour milk products from camel milk contribute to the normalization of the pancreas’ functionality, intestines, and liver; moreover, it encourages the bodily nervous system and increases human immunity to various infectious diseases. Different scientists [18, 19] saw more incredible health benefits to include products from camel milk into a diet for those with chronic digestive, cardiovascular, nervous, immune system skin diseases, and diabetes. These products positively enhanced the stomach’s excretory functionality and improved the absorption of proteins, fats, and carbohydrates. The comparative study recommended that the ordinary, everyday consumption of 200 ml drinking product decreases toxins in the body and improves the immune system. It must be noted that the nutritional value and therapeutic power of a fermented camel milk product depend on the availability of easily digestible proteins (albumen), antibiotics, vitamins, minerals, ethyl alcohol, which are produced by the fermenting microorganisms as the specific fat content, amino acids [20–22]. Besides these benefits, it has demerits too. Camel milk coagulation is lower than cow’s milk. Therefore, it isn’t very easy to produce curd mass or thickened yogurt or even cheese from it.

Following research works [23–28] were conducted on animal studies related to camel milk with the duration of experiment 5, 14, 21, 28, 31, 30 days, respectively. However, this research was carried out only on raw camel milk, and their main aim was to investigate the therapeutic effects of camel milk on various diseases. According to the above-indicated research, our study’s duration was selected to 17 days, including fasting hours, and it represents valuable research performed with developing a new curd mass product from camel milk, which matches gerodiet requirements. Moreover, this investigation’s main aim is assessment, identification, control, and change of physiological states of animals, which fed with new gerodiet product. Blood serum biochemical parameters (total protein (TP), cholesterol (Chol), glucose (Glu), albumin (Alb); the enzymatic activities of alanine aminotransferase (ALT), aspartate aminotransferase (AST), alkaline phosphatase (ALP), triglycerides (TG) and peptide toxicity (PT)) were taken as indicators of our investigation. To our knowledge, this is the first time when camel milk was suggested for use as a gerodiet product on its own and with nutritional supplements. The main reason for choosing curd mass is that it is suitable for older adults and has essential oral consumption properties, such as homogeneous consistency, soft texture, and light chewing. Besides, various functional foods and vegetable additives such as flaxseed flour (FF), celery root powder (CRP), and bee bread (BB) (ambrosia) have been added to increase the functionality of the curd mass (CM).

## 2. Materials and methods

### 2.1. Bioethical standards in animal husbandry and experimental protocol

A study on experimental animals conducted following the rules for the maintenance and care of laboratory rodents and rabbits, described in "Guidance on Animal Welfare Bodies and National Committees" of the Eurasian Commission (Directive 2010/63/EU). The official protocol (№1, from 06.01.2020) for animal experiments approved by the Local Ethics Committee of the Institute of humans and Animal Physiology of the Scientific Committee of the Ministry of Education and Science of the Republic Kazakhstan.

### 2.2. Reagents

Alkaline phosphatase (5x20 ml), albumin (1x250 ml), cholesterol (10x50 ml); glucose (1x500 ml), alanine aminotransferase (1x200 ml), aspartate aminotransferase (1x200 ml), total protein (10x50 ml), triglycerides (4x50 ml) standard specific kits were obtained from Biosystems S.A. (Spain) companies official supplier in Kazakhstan.

### 2.3. Preparation of CM

The local camel breeding farm "Daulet Beket" (Akshi, Karasay region, Almaty, Kazakhstan) kindly delivered camel milk (5.2% fat) for developing new product. For fermentation and coagulation, dry bacterial starter culture with composition *Lactococcus lactis subsp*. *lactis*, *Lactococcus lactis subsp*.*cremoris*, *Lactococcus lactis subsp*.*lactis biovar diacetylactis* (DVI, Vivo. Ltd. Kyiv, Ukraine) (the number of live bacteria in 1.0 g of the product—not less than 1×10 in the 9th degree CFU) and rennet (200 IMCU/mL, Chy-max PLUS Chr. Hansen Pty. Hoersholm, Denmark) were used. Calcium chloride and cheesecloth were also obtained from local supermarkets. Three various types of food and vegetable additives FF (300 g/p "Kompas zdorovya" Ltd. Novosibirsk, Russia), CRP (150 g, Windmill Health Products, West Caldwell, N.J., USA), and BB (250 g, "Bal-Ara" Ltd. Almaty, Kazakhstan) used for adding to CM. The obtained camel milk was filtered and standardized (fat content 1.3%±0.2) with skimmed milk (fat content 0.1%), heated to 55°C, homogenized, pasteurized (80–83°C, 50 sec or 60–65°C for 20–25 min), cooled to 45°C, then the starter culture, calcium chloride (after properly adjusting pH 6) and rennet added. Coagulation running for three h, and a soft clot formed (pH 4.6–4.7), after which the lump was cut and heated to 55°C and kept at this temperature until the serum was separated. Subsequently, before adequately draining, the curd was washed with cold water, poured into a colander lined with gauze cloth, and left for 5 h at ambient temperature under sterilized conditions. Curd mass yield is divided into three parts. FF [29], CRP [30], and BB [31] were added to each part of the curd in an amount of 0.3%-0.4%/100 g, respectively, and mixed until the mass became homogeneous. CM was stored at −4°C and used to feed the rats. Before producing CM, it is necessary to evaluate the chemical content of the camel milk and the qualities of milk depends on various environmental and breed uniqueness. It is essential to work out the additives’ proportions, consider the milk’s storage conditions, its freshness, and composition when ripening and forming the CM. Specialists should examine BB products for carbohydrate composition. When over saturated with simple carbohydrates, the effect of this product will significantly decrease.

### 2.4. Experimental animals, husbandry, and *in vivo* study design

**Fig 1:**
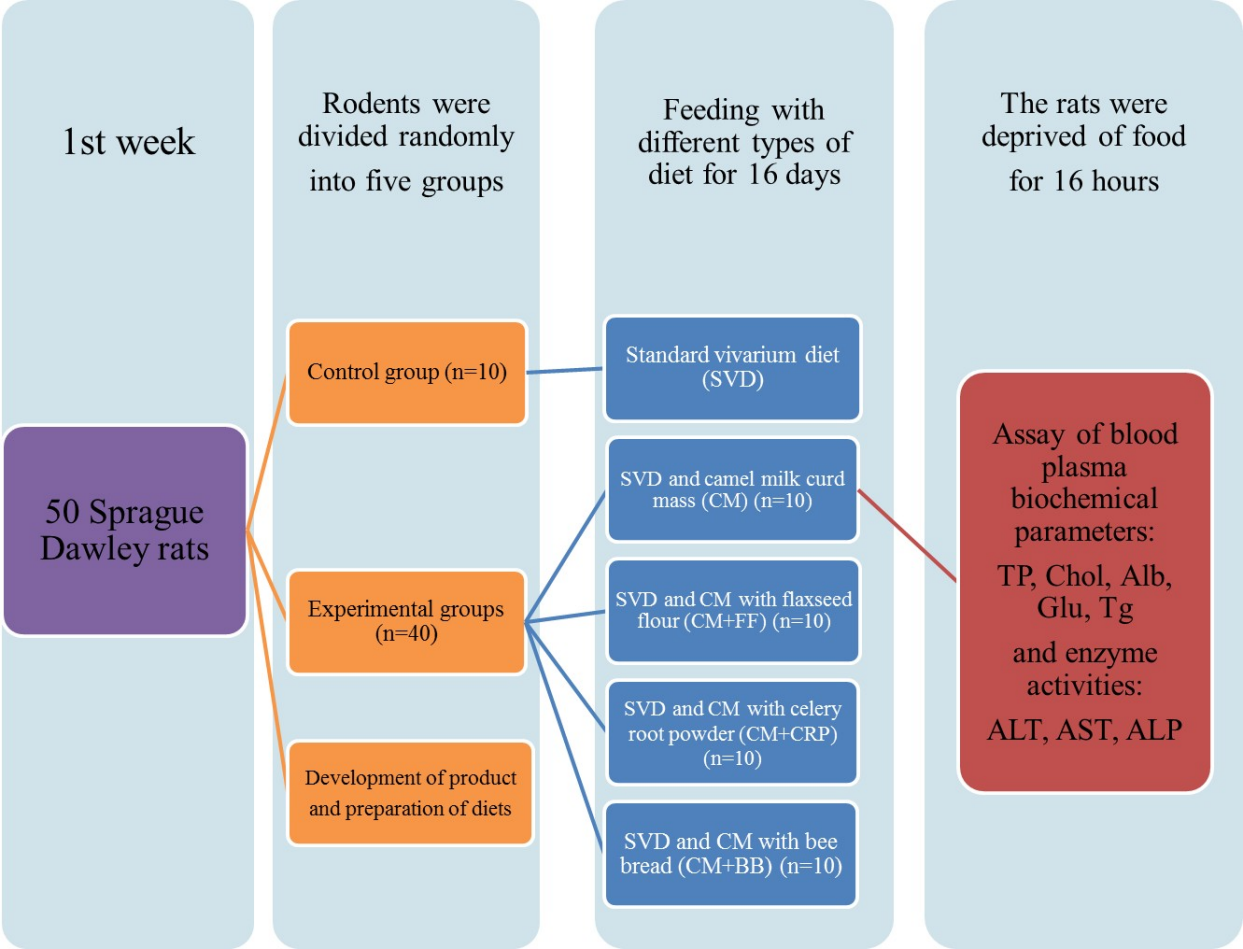
*In-vivo* study design.

For this experiment (see in-vivo study design on Fig 1), a total of fifty healthy Sprague-Dawley (SD) breed male rats weighing 302.8±0.05 g on average was used. The initial weight of animals can be seen in Fig 1. The research group obtained animals from Kazakh Scientific Centre for Quarantine and Zoonotic Infections (Almaty, Kazakhstan). Rats were aged from 12 to 14 months (rat age to human age ratio 30–35 years) [32]. The key reason for choosing rats at this age is adequately explained with some specific issues reasonably relating to the aged rat, which susceptible to various diseases, analogous to geriatric canine and feline ones [33]. In some cases, they do not live till the end of the experiment. After receiving current research data, we are planning to conduct a similar survey of aged rats as a continuation of this study. The rats were comfortably housed in metal—plastic cages (three animals per cell) and adequately kept in an air-conditioned animal room at 21±2°C temperature. Relative humidity 55% and 12/12 h light/dark period and the rats were kept there for the whole investigation period, i.e., 17 days. The study group provided animals’ unrestricted daily access to food and drinking water. Throughout this specific period, rat bedding was routinely turned over and cleaned daily to maintain good hygiene.

### 2.5. Groups and diets

Rodents were randomly divided into five groups (n = 10 per group). For this study, parallel-arm, the five-group design was used. The rats were subjected to a 1-week adaptation period on a standard diet. Before and during experiments, all animals precisely correspond to regular rodent feed (Nuvilab CR1s, Nuvital SA, Colombo-PR, Brazil), which consisted of 22% protein, 1.3±0.2% fat, 4% crude fibre, which corresponded to energy values of 290 kcal/100 g [33]. Additionally, four groups were fed in the morning (3 g/each) with diverse CM types for 16 days. The effective dose of various CM types was 1.0 g/100 g body weight/day. This consumption is approximately equivalent to the older adults’ consumption of 120 g ricotta cheese per day (typically depending on body weight mass), the texture of which is similar to CM (Australian Dietary Guidelines, 2020). The group content and suggested diet are presented in Table 1.

**Table 1:** Group name and diet content of Sprague-Dawley (SD) breed rats.

Group № (n = 10)	Main daily diet	Addition to the main diet
I–control	Standard vivarium (*ad libitum*)	-
II		Curd mass**
III		Curd mass and flaxseed flour^#^
IV		Curd mass with celery root powder^#^
V		Curd mass with bee bread (*ambrosia*)^#^

*Chemical content presented above.

**CM– 1.0g/100g of body weight animals.

^#^addition of FF, CRP, and BB to CM is 0.5%/kg product.

The diets energy, total number of proteins, fat, and carbohydrates are shown in Table 2.

**Table 2:** The energy, the total number of proteins, fat, and carbohydrates of the diets per 100 g.

Indicators	Groups				
	I	II	III	IV	V
Energy, kcal/100 g	290	62.2	74.3	63.45	74.27
Protein, g/100 g	22±0.1	3.18±0.25	4.26±0.3	3.22 ± 0.05	4.24±0.2
Fat, g/100 g	1.3±0.2	1.3±0.1	1.6±0.2	1.3 ± 0.09	1.4 ± 0.1
Carbohydrates, g/100 g	4±0.5	6.73±0.5	7±0.8	6.95±0.2	7.2±0.2

### 2.6. Dissection of rats

After 16 days of feeding experiments, the rats were deprived of food for 16 h, weighed, anesthetized with CO2, and sacrificed. According to the appropriate methodology adequately described by specific literature [34], all exposed rats’ dissection was carefully conducted. Only the central organ systems were observed. Descriptions of the musculature and skeleton were not provided. Whole blood was collected (with heparin as an anticoagulant, 2–3 u/ml), and serum was obtained by centrifugation at 1500 rpm for 10 min at room temperature (RT) (EBA 20, Hettich Lab Technology, Germany) to separate the blood into upper and lower layers. The serum was then accepted as a supernatant and refrigerated at −40°C for subsequent estimation of biochemical parameters.

### 2.7. Assay for serum biochemical parameters by automatic analyzer

Blood plasma TP, Chol, Glu, Alb, ALT, AST, ALP, and TG assayed using an A25 automatic chemistry analyzer (Biosystems SA, Barcelona, Catalunya, Spain), according to the manufacturer’s instructions.

### 2.8. Methodology of identification of peptide toxicity

The PT determination was conducted by advanced method [35]; in brief, 1.0 ml of blood serum carefully placed in a centrifuge tube, and a 10% TCA solution of 0.5 ml was added. Then, it was thoroughly mixed, and after 5 min, centrifuged for 30 min at 3000 rpm. Subsequently, 0.5 ml of the supernatant was transferred correctly to a tube with 4.5 ml of distilled water. Spectrophotometric measurements (Cecil, 1000 series, Cecil Instrumentation Services Ltd. Cambridge, UK) were conducted at a specific wavelength of 0.254 nm in a 1 cm cuvette against distilled water after carefully mixing.

### 2.9. Statistical analysis

Statistical analyses of all received data were conducted correctly using the Statistica 6.1 PL software (StatSoft, Inc.) and statistically processed in Microsoft Excel. ANOVA one-way analysis of variance test was used to determine the sample’s p-value dataset and identify whether any statistically significant differences exist. Tukey-Kramer’s HSD pairwise comparison for the inter group and Dunnett’s equal samples (DCV) test for comparison treatment groups against a control group mean was used to establish statistically significant differences. Given values are means ± standard deviation (SD). The statistical significance level has been set at p≤0.05.

## 3. Results

### 3.1 Body mass index changes

**Fig 2:**
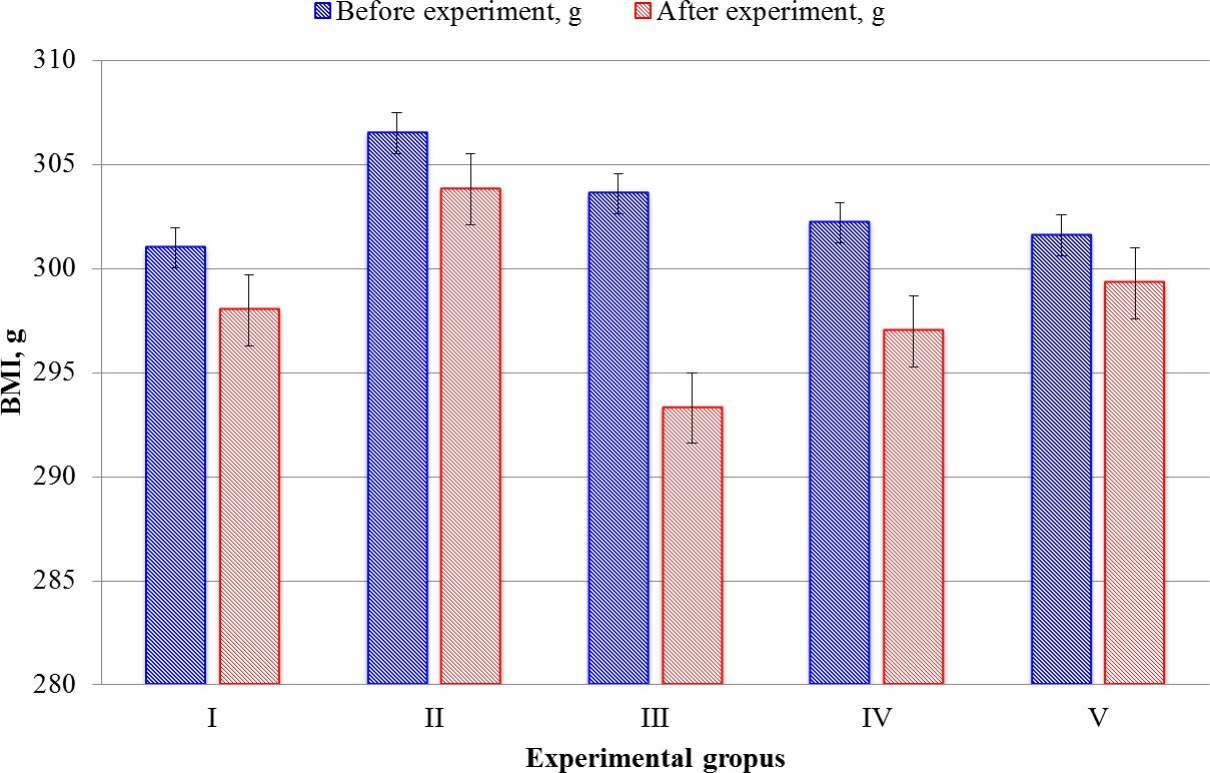
Comparing the product effect on changes in body weight of all groups before and after feeding, g. Data represent Mean ± SD. *Mean difference is significant at (α≤0.05) compared to the first day as determined by software at the end of the experiment. ANOVA’s P-value is 0.53, Qs is lower critical values associated with the Studentized Range Distribution Qa– 5.673, and SE is 2.78.

Over the 16 days of the experiments, the rats had regular solid food intake, including experimental product, and the body weight of each group was different compare to each other. CM’s daily intake was not different among the treatment groups (3g for each animal CM and its mixes; p<0.05). The weight changes (average) of the animals can be seen in Fig 2.

Calculations of received results by Tukey–Kramer’s test given in Excel sheet as S1 File. The absolute difference (AD) by Tukey–Kramer’s test control groups versus groups II, III, IV, and V are 5.65, 1.05, 0.1, and 0.95, respectively. Simultaneously, group II AD compared with other groups, except the control group was 6.77, 5.55, and 4.7. However, group III data versus groups IV and V were low, and the same scenario was with group IV to group V. Group I initial body weight to the treatment group slightly dropped to approximately 1% of the considerable weight measured on the first day of the experiment. The animal weight in group II ranged around 306.5 g before feeding with CM. However, after feeding, it decreased by ~1%. The considerable percentage of modest weight loss of animals in group III was the most significant than initial weights (w.a.) > than 5% drop. There was undoubtedly a slight decrease in body weight of approximately 3.7% and 5% from the initial value for IV and V groups, respectively. The comprehensive data’s adequate reliability compared with the results obtained before and after feeding is p≤0.05.

### 3.2 Rat dissection

The completed autopsy did not detect morphological changes in the structure of internal organs. The mucous membranes of the oral cavity, throat, and esophagus were pink; the authors did not ensure lateral inclusions. The mucous membrane of the stomach and intestines were whitish-gray-pink. The pancreas was healthy, and the liver was red-brown, elastic, and without any notable inclusions. The kidneys were gray-brown, flexible; the renal pelvis was clean, empty, and without additions and deposits. Lungs and heart were without specific pathology. Tissues in common were in an elastic state, springy consistency, and the blood is dark red. Its coagulation is normal. Predominantly, researchers did not detect pathological changes in the tissue and organs at all rats’ dissection. Rats were healthy, mature, visually internal organs were in good condition.

### 3.3 Serum biochemical parameters

**Fig 3:**
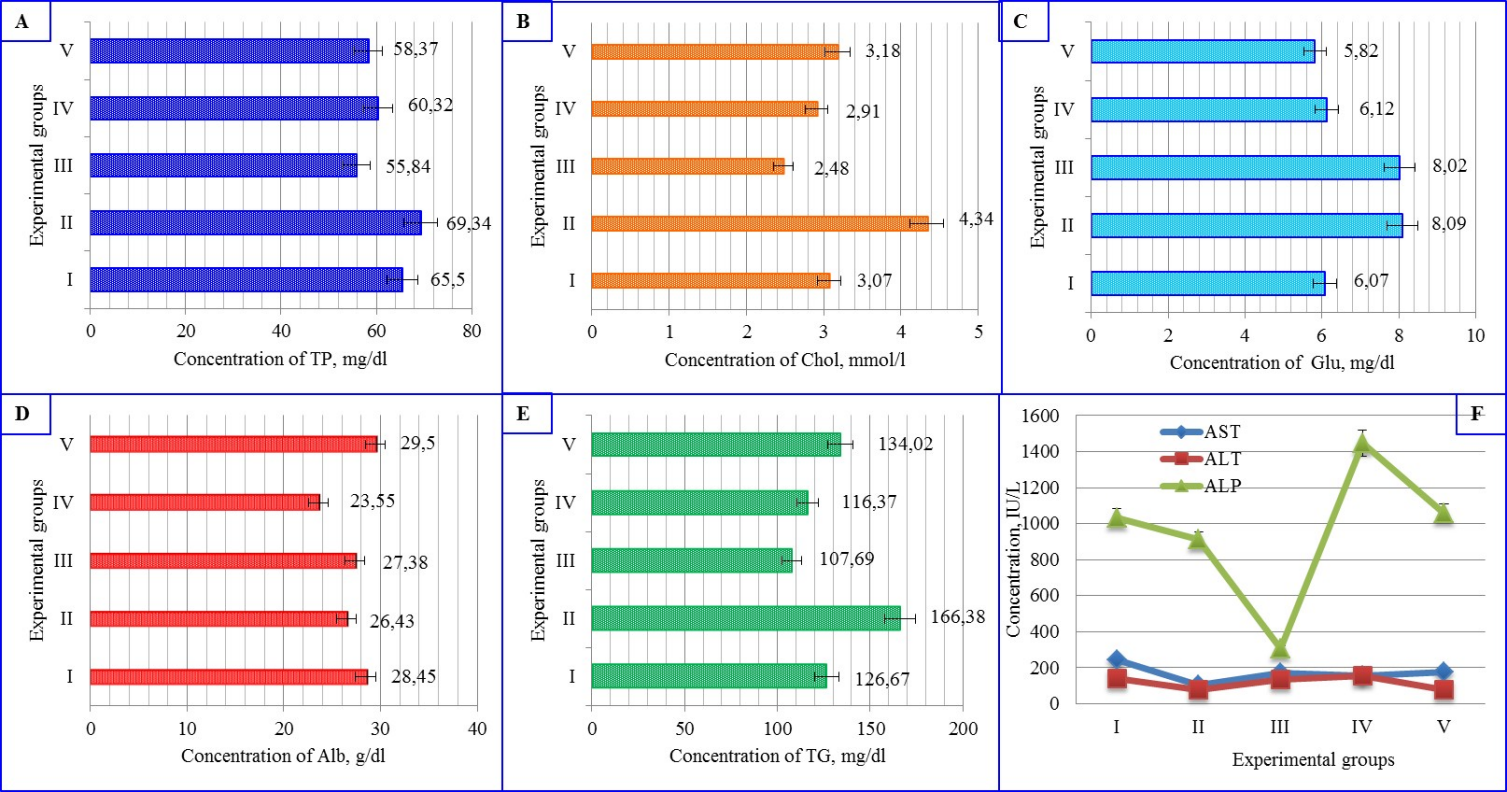
The results of assay of blood serum biochemical parameters after experiment. Groups: I = control, II = curd mass, III = curd mass mixed with flaxseed flour, IV = curd mass mixed with celery root powder, V = curd mass mixed with bee bread. (A) TP decreased in groups III, IV, V, but increased in group II compared to group I. (B) Chol level grew up in group II; in groups III and IV, it dropped; however, in group V slightly raised a group I results. (C) Glu raised in II and III, IV groups compare to I group; still, V group results show slightly decreasing; (D) Alb concentration decreased in group IV, yet in group V, it increased significantly than in group I results. Meanwhile, it changed groups II and III results with less percentage. (E) TG level dropped slightly in groups III and IV; however, in groups II, V it expanded. (F) AST levels in all blood plasma samples remained reduced by 30%-37% compared with the group I data. During the experiment, additives demonstrated a significant decrease in ALT in group III by 46% and increased in the IV and V groups up to 5% and 13%, respectively. Groups II, III, IV De Ritis ratio decreased to 25%-27%, and group IV increased to 44% compared to group I results. *Values are given as mean ± SD for a total of 50 animals. p<0, 05 versus mice fed regular diet.

Dunnett’s test for equal samples was used to evaluate are there any significance among received data from the automatic A25 analyzer, and the treatment group’s results were compared with control group results separately. Fig 3 illustrated the received results of biochemical parameters of blood serum for each group. Completed calculations by Dunnett’s formula presented in S1 File.

#### 3.3.1. Total protein

Our received results presented that, there were significant differences of the TP content in all groups’ results. Group II (69.34 mg/dl) data was higher than the control group (65.5 mg/dl). However, groups III (55.84 mg/dl), IV (60.32 mg/dl), V (58.37 mg/dl) were lower than group I result (Fig 3, S2A in S1 File).

The absolute difference (AD) in comparison pair of groups I versus groups II, III, IV, V were 3.84, 9.66. 5.18. 7.13, respectively, and Dunnett’s Critical value (DCV) was 0.44 for all groups. These received results rejected H˳, accepted Hα, which concluded that the results of groups II, III, IV, V are not equal with the control group (I), and their data are significant (α<0.05).

#### 3.3.2. Cholesterol

The Chol level in groups III (2.48 mmol/l), IV (2.91 mmol/l), V (3.18 mmol/l) was low and group II (4.34 mmol/l) data was higher than control group (3.07 mmol/l) (Fig 3, S2B in S1 File).

The results of group II and III compared to group I identified significant effects of AD to DCV, and Hα accepted for these groups; nevertheless, for other groups III, IV this hypothesis rejected, and their results presented as a low AD than DCV, which means they are not significant.

#### 3.3.3. Glucose

Interestingly on the fact that the Glu level in groups II (8.09 mg/dl), III (8.02 mg/dl) and IV (6.12 mg/dl), were above control groups’ result (6.07 mg/dl), while group V (5.82 mg/dl), demonstrated less amount (Fig 3, S2C in S1 File).

Comparing group I to group II showed 2.02 AD, group II– 1.95 AD, and group V– 0.25AD. However, the comparison pair of groups I versus group IV presented low AD at 0.05. DCV was 0.07 for all groups. These calculations demonstrated that the Glu level of groups II, III, IV versus group I is significant, and their data not equal with control group results. At the same point, comparison pairs of groups I to group IV do not have significance, which means their products are identical.

#### 3.3.4. Albumin

The Alb content results in all treatment groups (II, III, IV, and V) compared to group I was significant. Groups II (26.43 g/dl), III (27.38 g/dl), IV (23.55 g/dl) demonstrated lower value and group V (29.5 g/dl) was higher than control group (28.45 g/dl) (Fig 3, S2D in S1 File).

Each group’s AD was 2.02, 1.07, 4.09, and 1.09, which were higher than DCV—0.49. Dunnett’s test for equal samples accepted the Hα, where control groups result not identical with treatment group data.

#### 3.3.5. Triglycerides

TG’s rank dropped to in the III (107.69 mg/dl) and IV (116.37 mg/dl) groups. It increased in groups II (166.38 mg/dl) and V (134.02 mg/dl) (Fig 3, S2E in S1 File).

The calculation of TG results from insignificance by Dunnett’s test in all groups was higher than the control group (I). AD presented as 39.71, 18. 98, 10.3, 7.35, respectively, and DCV were at 0.52. These calculations approved Hα and accepted that the control group’s result not similar to the treatment group’s results.

The control and experimental group’s results of enzymatic activity comparison were also calculated with Dunnett’s test for equal samples. Completed calculations by Dunnett’s formula presented in S1 File.

#### 3.3.6. Aspartate aminotransferase

In all cases, the AST concentration decreased on average by 37% compared with the group I, which indicates a reduced level of damage to the heart muscle and cardiomyocytes (Fig 3, S3 in S1 File). AST data were significant in comparison pair groups I versus II, III and the AD showed as a 2.02, 1.95, respectively, which presented that their results are not equal. Meanwhile, groups IV and V results did not identify significance, and the AD demonstrated as a 0.05, 0.25. DCV is set at 0.63.

#### 3.3.7. Alanine aminotransferase

During the experiment, additives demonstrated a significant decrease in ALT in group III by 46% and increased in the IV and V groups up to 5% and 13%, respectively (Fig 3, S3 in S1 File).

ALT results approved Hα, which explains that the control group (I) result not equal with all treatment groups in comparison pairs. AD presented for group II—60.14, III- 6.31, IV—17.8, V—63.9 in pairs with group I. DCV calculated as 0.73.

#### 3.3.8. Alkaline phosphatase

Similar results were identified with ALP as described above enzymes. Comparison pairs of groups I versus groups II, III, IV, V were calculated as 121.21, 725.41, 413.29, 25.49, respectively, and DCV was 0.62. These results concluded that received data are significant, and data of all groups not equal to group I. The ALP concentration fluctuated within the control values in group V. However, its level in group IV increased up to 40%, and in group III decreased to 70% (Fig 3, S3 in S1 File).

AST/ALT ratio of group I was 1.76 U/L; but, the group II result decreased to 25%, and the group III reduced to 27.5%, whereas the results of group IV reveal the negligible proportion of AST and ALT varied with a minimum physiological norm and much lower than III group data. This group’s results detect an increasing concentration of ALT to 13%, ALP to 40%; simultaneously AST decreased to 37%, which means that celery root powder causes changes in the functions of the liver and hepatocytes, but prevents myocardial damage. The De Ritis coefficient in group V exceeds the maximum physiological standard and group I data up to 30%. BB to CM’s addition revealed a significant effect, where ALT decreased by 46%, AST by 30%, ALP fluctuated within the control data levels. Group V results showed a high De Ritis ratio (AST/ALT) compared to group I result.

### Peptide toxicity of products

**Fig 4:**
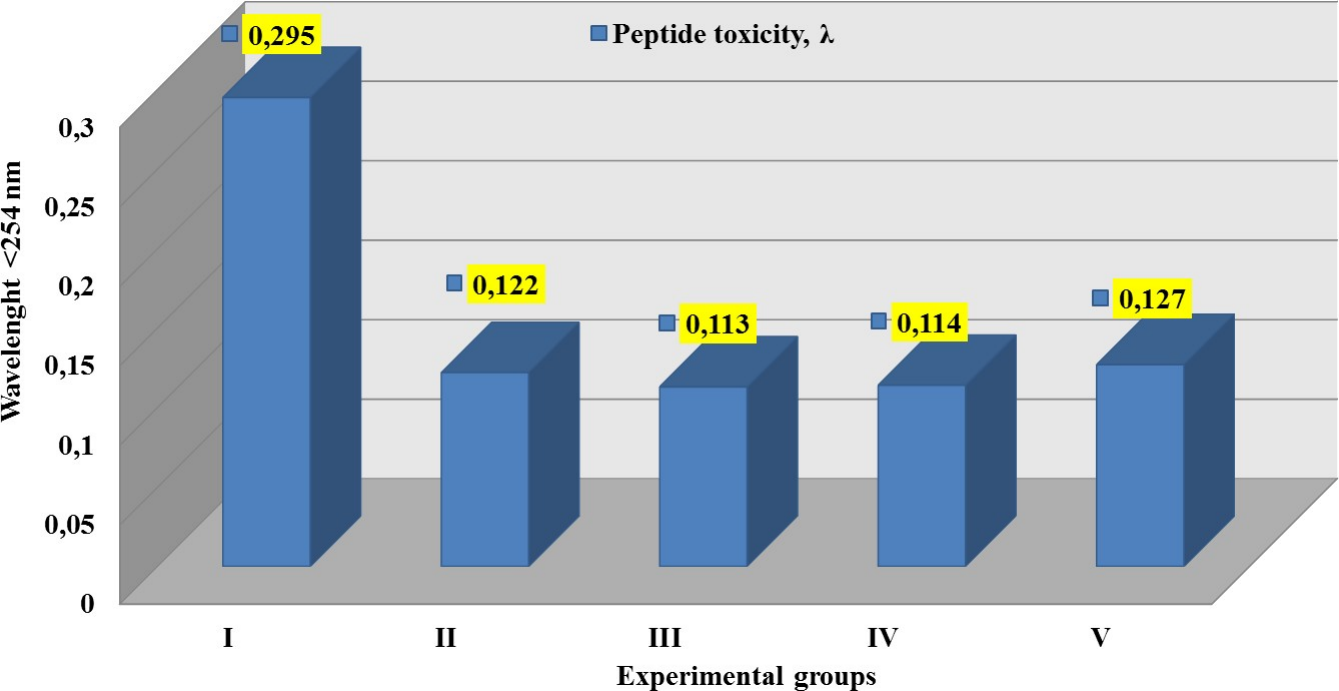
Results of peptide toxicity of each diet. Values are given as mean ± SD for a total of 50 animals. Control group (I) - 0.295 nm, Curd mass (II)– 0.122 nm, Curd mass with flaxseed flour (III)– 0.113 nm, curd mass with celery root powder (IV) - 0.114 nm, Curd mass with bee bread (V)– 0.127 nm. There were decreasing in PT of all samples compared to group I. Groups II and III results presented a lower PT with some fluctuation compared to other group results. The group III results higher than II to 0.005 nm. The group I shows superior results, but lower than main PT requirements.

Received data sufficiently illustrated a sharply decreasing diet PT by 2.0–2.5 considerable times compared with the group I, as displayed in Fig 4.

It must be prominently mentioned that the visible results of groups III and IV were similar to a 0.001% difference; however, the group II and V results indicated a noticeable increase of 7%-10% more than the previous groups lower to 60% than group I result.

By Dunnetts’s test for equal samples, PT results in comparison pairs of the control group with other treatment groups showed high significance; however, DCV set as 0 by calculation and AD for paired groups was 0.173, 0.182, 0.181, and 0.168, respectively.

## 4. Discussion

In this study, 16 days of treatment with camel milk curd mass given as a pure and flaxseed flour, celery root powder, and bee bread in an experimental model of Sprague-Dawley rats resulted in improvement of metabolism, which was manifested by a reduction in Chol, ALP. It was also accompanied by increased carbohydrate-fat metabolism, which suggested that it can help prevent low-density lipoprotein plaques in the arteries.

After 16 h of deprivation, the average percentage of all groups’ modest weight loss after 17 days was 3.14%, and the maximum presents 5% in the two groups. 16 h of deprivation reported here similar with previous studies [36–39]. Other studies report a 15% higher average weight loss in exposed Wistar rats after 16 h of deprivation [40]. In different rat strains and unexpectedly more significant reduction of up to 18% after 24 h of fasting was reported [41–43].

Regarding the moderate weight loss observed in this study, compensatory water intake can be excluded. Various studies have shown that modest weight loss during periods of deprivation of 3 and 4 days is accompanied by decreased water intake [40, 44].

Another critical variable could be the animal’s age, with younger rats losing a more significant percentage of body weight than older rats. This was described in research work [45], where more immature rats lost 29% of body weight in 24 h of deprivation of food. Therefore, we assume that our weight loss data do not exceed the allowable weight loss limit set by the regulatory body and the animal ethics committee. What’s more, it can be reasonably supposed that the short-term consumption of pure CM or with above-selected food and vegetable additives can prevent obesity and smooth weight loss. A similar conclusion was described in work, which results concluded that fermented camel milk could provide a beneficial effect on the inflammation associated with obesity [46]. Even though the above-listed additives offer enormous health benefits [47–57], they have not been considered an add-on to camel milk until recently.

We have demonstrated that a pure CM–fed group (II) had higher TP, Chol, Glu, TG and lower plasma Alb, enzymes AST, ALT, ALP compare to the control group; even more, the PT of this group was two times lower versus group I. Also significant differences in CM plus FF–fed group (III), TP, Chol, Alb, TG, and AST, Alt, ALP were decreased; however, Glu level increased compared to group I. The identified PT of diet also was lower than control group results. Group II fed with CM plus CRP demonstrated changes versus the control group. The levels of TP, Chol, Alb, TG, and AST dropped significantly compared to group I. Nevertheless, the Glu, ALT, ALP was higher than the control group. Meanwhile, diet PT still was low as identified in other groups compared to group I. A similar scenario was with the group fed with CM plus BB, where TP, Glu, and AST, ALP dropped slightly, but levels of Chol, Alb, TG, and ALP increased. However, diet PT was lower than the control group and higher than the other three groups. These prime factors were also presented in other study results [58] where the inclusion of 20% flaxseed in diets of rats’ decreased total plasma Chol, TG, and low-density lipoprotein (LDL) Chol by 21, 34, and 23%, respectively [59].

Our received data from group V found that BB has rich biochemical and antioxidant content and has many therapeutic properties like antimicrobial, antitumoral antibacterial, immunomodulatory, and anti-inflammatory properties [60]. Different research evaluated the effect of BB, administered as BB seed and mice [61]. Mice were divided into three groups and were orally administered BB (250 mg/kg BW) for 21 days. It was established that the level of lipid peroxidation (LPO) decreased in the group in BB was administered compared to the control. The data of post-treatment analysis of patients’ blood samples suggest BB has a positive effect on the immune system and helps adjust the lipid metabolic disorders of older adults [62, 63].

The identical results have been observed in the following research works regarding celery. Scientists presented the effects of celery on serum lipids of mice [64]. Mice fed high-fat meals, and the results showed a significant decrease in low-density lipoprotein and cholesterol, which did not affect Very-low-density lipoproteins and "good cholesterol."

It must be observed that BB, CRP did not use with milk products, and the above-mentioned studies are conducted only on them without any mixing.

The following research works have sufficiently demonstrated the profound effect of camel milk on serum enzymatic activity. The research group found treatment with camel milk could promptly suppress the noticeable increase of serums AST and ALT activity induced by gentamicin treatment in rats [65]. This finding implies that CM can repair and adequately protect liver tissue through membrane-stabilizing and leakage prevention of intracellular enzymes.

Various research works [27, 28, 66, 67] sufficiently indicated the effect of camel milk on the animal models. Their qualitative analyses adequately describe raw camel milk and do not connect with camel milk products’ potential impact on the organism. In this manner, our PT assay data results can reasonably assume that CM and its mixtures are more beneficial in sufficiently reducing the organism’s peptide toxicity and can prevent sedimentation processes. Results indicate a moderate level of accumulation of peroxide products in the blood, which prevents damage to the body’s cells and tissues with the prolonged intakes of a fermented milk product. The resulting significant effect is associated with the fermented milk product’s superior antioxidant capacity in a mono-diet and with food additives, protects the molecules of cell membrane components from oxidation, and, therefore, preserves their activity and the balance of oxidative activity in the blood.

The blood plasma samples of all groups show the fast digestibility of CM by the body’s cells; moreover, it gains a preventive effect at the cellular level and prevents toxic damage to heart and liver cells. Many of our key findings are appropriately attributed to the fundamental nature of the camel milk’s invaluable addition to its fermented status. The concomitant decrease of serum lipids and a lesser increase in weight and BMI of the animals (though non-significant) may have mediated their liver enzymes’ favorable alterations. All the possible results and side effects of CM and their explanatory mechanisms must be investigated in further studies. However, there is limited valuable information for properly using BB and CRP as a geroprotector or with milk products; nevertheless, assigning to our direct results, we can conclude that consumers must carefully keep a few specific recommendations. Granting to our accepted data and other research works, we can reasonably suggest that BB could be used as a food additive with CM with some recomendations in daily consumption dosage. According to these results, we can assume that BB with CM significantly impacts liver functionality. Simultaneously, CRP with CM could decrease the preventive capability of the liver. Our research results indicate that effective use of pure CM can slightly increase Chol and change carbohydrate-fat metabolism. FF with CM can be ordinarily used for lowering blood Chol and Glu. The lack of well-designed trials limits the current evidence to decrease the influence of biases.

## 5. Conclusion

In conclusion, the experimental results showed that pure cottage mass and its diverse mixes may be adequately considered as a daily dietary supplement. Moreover, results presented that the curd mass with flaxseed flour can reduce weight and positively enhance carbohydrate-fat metabolism. At the same time, curd mass with celery root powder can prevent low-density lipoprotein plaques in the arteries. In addition, the curd mass moderate preventive effect with bee bread on the liver’s functions and heart. The research results showed that cottage mass could be used as a pure or with additives. For the future, we are strategically planning to continue our research work thoughtfully in the appropriate order on elderly healthy rats based on this practical experiment’s results.

## Supporting information

S1 File Following supporting information attached to this manuscript: *Calculations of BMI changes before and after experiment by Tukey-Kramer’s inter group test; * Calculations of TP, Chol, Glu, Alb, TG, AST, ALT, ALP, PT significance in control group results against other experimental groups by Dunnett’s equal samples test. (XLSX)
